# Training machine learning algorithms for automatic facial coding: The role of emotional facial expressions’ prototypicality

**DOI:** 10.1371/journal.pone.0281309

**Published:** 2023-02-10

**Authors:** Björn Büdenbender, Tim T. A. Höfling, Antje B. M. Gerdes, Georg W. Alpers

**Affiliations:** Department of Psychology, School of Social Sciences, University of Mannheim, Mannheim, Germany; Sejong University, REPUBLIC OF KOREA

## Abstract

Automatic facial coding (AFC) is a promising new research tool to efficiently analyze emotional facial expressions. AFC is based on machine learning procedures to infer emotion categorization from facial movements (i.e., Action Units). State-of-the-art AFC accurately classifies intense and prototypical facial expressions, whereas it is less accurate for non-prototypical and less intense facial expressions. A potential reason might be that AFC is typically trained with standardized and prototypical facial expression inventories. Because AFC would be useful to analyze less prototypical research material as well, we set out to determine the role of prototypicality in the training material. We trained established machine learning algorithms either with standardized expressions from widely used research inventories or with unstandardized emotional facial expressions obtained in a typical laboratory setting and tested them on identical or cross-over material. All machine learning models’ accuracies were comparable when trained and tested with held-out dataset from the same dataset (acc. = [83.4% to 92.5%]). Strikingly, we found a substantial drop in accuracies for models trained with the highly prototypical standardized dataset when tested in the unstandardized dataset (acc. = [52.8%; 69.8%]). However, when they were trained with unstandardized expressions and tested with standardized datasets, accuracies held up (acc. = [82.7%; 92.5%]). These findings demonstrate a strong impact of the training material’s prototypicality on AFC’s ability to classify emotional faces. Because AFC would be useful for analyzing emotional facial expressions in research or even naturalistic scenarios, future developments should include more naturalistic facial expressions for training. This approach will improve the generalizability of AFC to encode more naturalistic facial expressions and increase robustness for future applications of this promising technology.

## Introduction

Automatic Facial Coding (AFC) is now ubiquitous and has made great strides in emotion recognition of facial expressions. Such technology is promising because facial expressions are highly relevant for social interactions and carry information about the emotional state of an individual [1, 2]. Thus, assessing and quantifying emotional facial expressions is a primary objective in emotion research [3]. AFC follows the logic of the best established observational system, the Facial Action Coding System (FACS; [4]), which requires manual assignment of facial movements (i.e., Action Unit; AU) by human expert observers. AFC is now promising to accelerate the process of coding facial activities, especially for large samples or large amounts of data making the method more feasible for naturalistic assessments in and outside the laboratory. Because it is mainly unclear how classifiers are trained in AFC systems which are typically proprietary, we investigate the impact of training materials’ prototypicality and the consequent influence on classification performance.

### AFC of standardized facial expressions

AFC software, as of today, typically entails two steps of analyzing faces [2, 5]: In the first step, physical properties of the pictures (here, AU features) are extracted, and in the second step, this information is integrated in terms of emotion categories. Regarding the first step, there is evidence of a high agreement between AFC and experienced human FACS coders [6–8] comparable to the inter-rater reliabilities between human FACS coders [9]. In the second step of AFC, developers face the challenge of deciding which and how many emotion categories should be predicted. Also, they need to choose the specific machine learning algorithm for the AFC software (for overviews, see [10, 11]). The so-called basic emotions (i.e., joy, sadness, anger, disgust, fear, and surprise) are the most extensively researched facial expression categories (e.g., [12]). They typically serve as predefined emotion categories for AFC’s supervised learning. Another challenge for new AFC tools is the fact that machine learning algorithms require a large amount of stimulus material (i.e., emotional faces) for their training, which is critical for the stability and generalizability of such systems. To access such datasets, AFC developers typically rely on standardized inventories of highly prototypical emotional facial expressions.

There is abundant evidence that AFC performs impressively in categorizing pictures from standardized inventories, typically comprising intense and prototypical emotional facial expressions (see Table 1). AFC classifies facial expressions of basic emotions with a high accuracy in standardized research inventories, which is a robust pattern for static picture inventories and dynamic video inventories [13–18]. However, decoding the facial expressions of skilled actors exaggerating certain emotions might not always correspond to actual emotional facial reactions that occur spontaneously. Hence, the exclusive use of such material for the development and validation of machine learning procedures can be deceiving in terms of the upper limit performance, in particular for these six basic emotions. Thus, it is essential to validate the sensitivity and specificity of AFC approaches for less intense and less prototypical facial expressions.

**Table 1:** Benchmark of automatic facial coding in A) prototypical and B) non-prototypical stimuli.

Authors	Classifier	Accuracy
**A) Prototypical**	Azure	.81
Küntzler et al. (2021) [19]		
Küntzler et al. (2021) [19]	Face++	.79
Küntzler et al. (2021) [19]	FR	.97
Lewinski et al. (2014) [18]	FR	.88
Skiendziel et al. (2019) [7]	FR	.80
Stöckli et al. (2018) [20]	AFFDEX	.73
Stöckli et al. (2018) [20]	FACET	.99
Yitzhak et al. (2017) [15]	CERT	.88
**Mean**		**.86**
**B) Non-Prototypical**	Azure	.57
Küntzler et al. (2021) [19]		
Küntzler et al. (2021) [19]	Face++	.32
Küntzler et al. (2021) [19]	FR	.31
Stöckli et al. (2018) [20]	AFFDEX	.55
Stöckli et al. (2018) [20]	FACET	.63
Stöckli et al. (2018) [20]	AFFDEX	.57
Stöckli et al. (2018) [20]	FACET	.67
Yitzhak et al. (2017) [15]	CERT	.21
**Mean**		**.48**

### AFC of unstandardized facial expressions

Regarding AFC performance in less standardized pictures, we recently demonstrated substantial differences in facial movement (AU activity) between highly standardized and prototypical facial expressions compared to unstandardized and less prototypical emotional faces [19, 21]. Consequently, AFC accuracy rates substantially decrease evaluated in less prototypical stimuli (see Table 1) when untrained participants are instructed to mimic or pose facial expressions, particularly for emotion categories like sadness or fear [15, 20, 22]. Consistent with these results, we found that AFC parameters of standardized inventories and unstandardized facial expressions from untrained participants in a typical laboratory setting substantially differ in the relative intensity of AU activity, the resulting AU profiles, and overall classification accuracies. Furthermore, the classification performance of AFC decreases if spontaneous facial responses toward emotional stimuli like scenes or faces are investigated [23, 24]. Hence, the validity of AFC to detect emotional facial expressions is further decreased compared to prototypical facial expressions from standardized inventories.

One potential mechanism that causes this gap in AFC performance between prototypical and non-prototypical facial expressions may be the common usage of prototypical emotional facial expressions for the training of new AFC systems. In the past, AFC systems heavily relied on highly standardized static and dynamic facial expression inventories to both train and test their developed machine learning models [25–37]. Hence, the decrease in the accuracy of AFC to detect less intense and non-prototypical emotional facial expressions in more naturalistic research settings may indicate an overfit of trained AFC machine learning models to detect prototypical facial expressions.

### Aims and overview

We set out to examine how the choice of specific material for training affects the accuracy of AFC machine learning algorithms to classify prototypical and non-prototypical emotional facial expressions. To this end, we used two datasets with pictures of emotional facial expressions (basic emotions: joy, sadness, anger, disgust, fear, and surprise) and extracted their AU intensity. One dataset comprises prototypical, standardized, and well-established research inventories (*standardized* dataset). The other comprises untrained study participants from a typical laboratory setting (*unstandardized* dataset), who were instructed to display emotional facial expressions. The aim was to investigate the influence of the prototypicality of training material on machine learning classification performance. To this end, we trained three machine learning algorithms based on the same set of AU parameters separately for both datasets (*standardized* vs. *unstandardized*) and tested their classification performance on both types of facial expressions. We expected a more accurate classification for the machine learning models trained and tested with matching datasets. Furthermore, we expect that the models which were trained with the *standardized* dataset would classify facial expressions from the *unstandardized* dataset less accurately. This study contributes to a better understanding of the inconsistent accuracies of AFC (see Table 1). Furthermore, it provides implications for the development and training procedure to approximate future AFC algorithms’ robustness and ecological validity.

## Materials and methods

### Datasets and facial expression analysis

The *standardized* dataset includes expressions from 69 women selected from widely used and standardized picture inventories (Karolinska Directed Emotional Faces, Warsaw Set of Emotional Facial Expression Pictures, Radboud Faces Database, [38–40]). The *unstandardized* dataset consists of expressions from 69 untrained female students who participated in an experiment in our laboratory (for details on the experimental procedure, see [21]). Participants were instructed to display emotional facial expressions cued by pictures of emotional facial expressions presented on a screen. Each dataset comprised pictures of 69 individuals who depicted posed facial expressions for the basic emotion categories joy, anger, surprise, sadness, disgust, fear, and neutral [41, 42].

Both datasets were processed with FaceReader (FR, Version 7.1, Noldus Information Technology) [43]. For each stimulus, the intensities of twenty Action Units were extracted (AU; AU01—Inner Brow Raiser, AU02—Outer Brow Raiser, AU04—Brow Lowerer, AU05—Upper Lid Raiser, AU06—Cheek Raiser, AU07—Lid Tightener, AU09—Nose Wrinkler, AU10—Upper Lid Raiser, AU15—Lip Corner Depressor, AU17—Chin Raiser, AU18—Lip Tightener, AU24—Lip Pressor, AU25—Lips Part, AU26—Jaw Drop, AU27—Mouth Stretch, AU43—Eyes Closed).

The FR classifies AU intensities with the following algorithmic pipeline [44, 45]: The face is located with a cascade classifier algorithm [46]. Textures of the face are normalized, and an active appearance model synthesizes a digital face model with over 500 location points [47]. Finally, compressed distance information is transmitted to an artificial neural network (ANN; [48]) that classifies the intensities of twenty AUs.

Two happy and one neutral picture from two actors in the *standardized* dataset were excluded because the FR did not reach convergence in the model fit, resulting in *N* = 480 observations. None were excluded from the *unstandardized* dataset (*N* = 483 observations).

The resulting AU activity scores for each emotional facial expression are predictors of our machine learning procedure, with the respective intended emotion as the prediction criterion. Both datasets are publicly available and completely anonymized and can be obtained from the https://madata.bib.uni-mannheim.de/327/ [49]. All lab participants contained in the *unstandardized* dataset provided written informed consent, and the experiment was approved by the University Mannheim Research Ethics Committee (EK Mannheim 09-3/2018) [21].

### Selected algorithms and hyperparameters

We investigated the effect of the prototypicality (manifested as differences in the AU activity [21]) of the *standardized* and the *unstandardized* dataset on the accuracy of the emotion classification with the following three machine learning algorithms: decision tree [50], random forest [51] and multi-layer perceptron [52]. We optimized hyperparameters with a resampling method (10-fold, grouped cross-validation, see “Machine learning procedure”), maximizing kappa.

The following hyperparameters were optimized: complexity (*cp*) for the decision trees, the number of trees (*ntree*) and variables considered at each decision node (*mtry*) in the random forest, and the number of neurons in the hidden-layer (*size*) for the multi-layer perceptron. Hyperparameter tuning and the training of the models were conducted with the caret R-package [53, 54]. All further hyperparameters of the algorithms were set to their default values [54]. A complete list of the R-packages used in the analyses is provided in the S1 Table.

#### Decision trees

We employed the rpart R-package [50] to train the decision tree. A decision tree is built by identifying the variable that bests splits the data into two groups (i.e., minimized impurity). This procedure is applied recursively to all generated subgroups and continued until the subgroup reaches a minimum size or no further improvement is possible. Such so-called greedy approaches will likely result in complex trees that will not generalize well on new data. To prevent overfitting, the resulting tree is thus, pruned by penalizing the number of terminal nodes. We tuned the complexity (*cp*) hyperparameter, which determines the minimum improvement necessary in a node split during the pruning process.

#### Random forest

For the random forest (RF), we used the *randomForest* R-package [51]. The RF is a bagging (i.e., bootstrapping and aggregating) ensemble learning method in which multiple independent unpruned decision trees are trained from bootstrapped samples, and their results are aggregated, in case of a classification problem, with a majority vote [55, 56]. Further, to build rather uncorrelated trees, only a random subset of all predictors in the dataset is considered at each decision node in the process of creating the trees. This procedure is supposed to make the RF robust against overfitting [55]. We optimized two hyperparameters: the number of variables considered for each decision node (*mtry*) and the number of trees in the forest (*ntree*). In order to perform an extensive grid search for both hyperparameters, we used a slight modification of the RF implementation in caret [51] to simultaneously optimize both hyperparameters (*mtry* and *ntree*).

#### Multilayer perceptron

We tested different numbers of hidden layers with no improvement in the model’s performance. Therefore, in the hyperparameter tuning phase, we only optimized the number of neurons in a single hidden layer (*size*) in order not to increase the risk of overfitting. The caret R-package [54] uses the RSNNS R-package [52] to train the multi-layer perceptron.

### Machine learning procedure and performance evaluation

**Fig 1:**
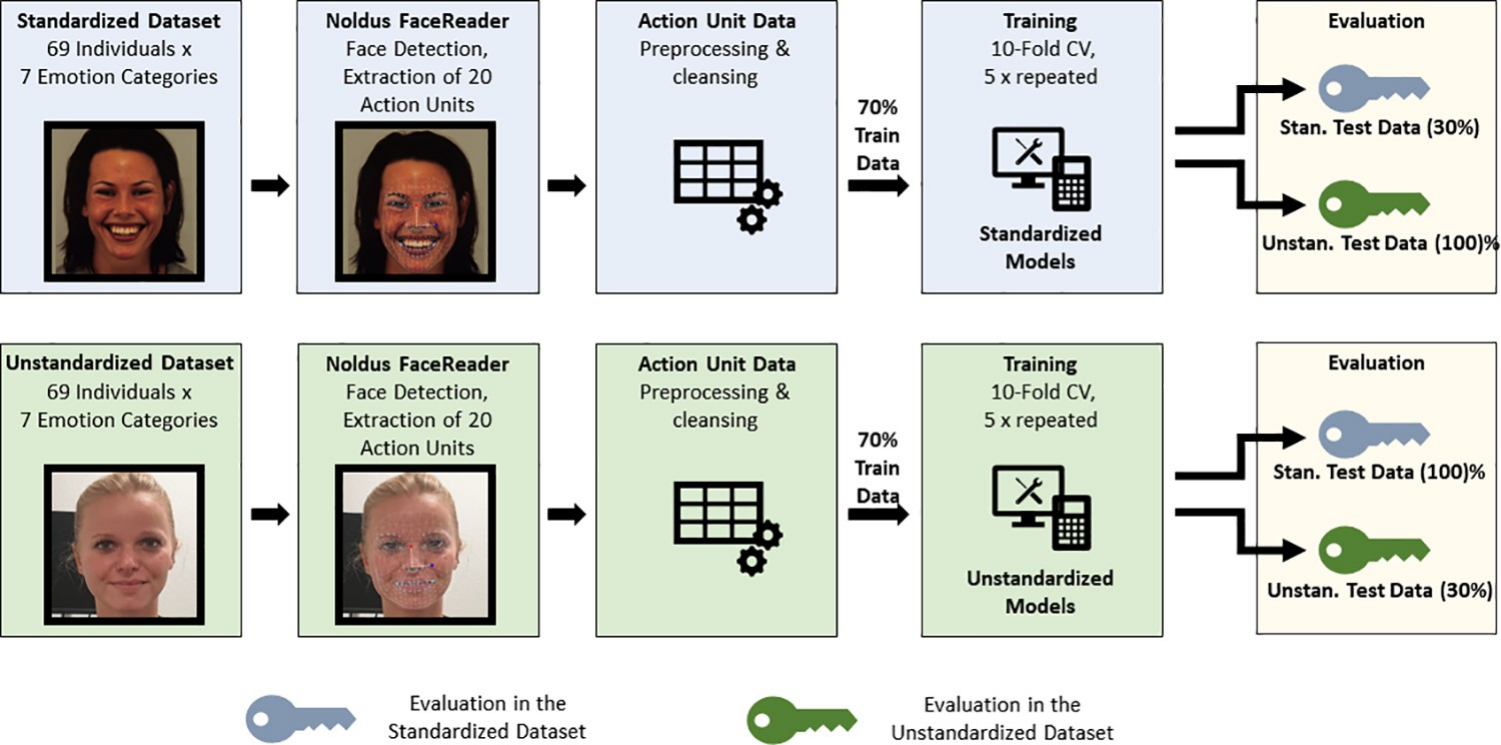
Machine learning pipeline. *Note*. Action Unit (AU) activity in both datasets was extracted with Noldus FaceReader (FR, Version 7.1). The training process for both datasets was identical: preprocessing, split into 70% training dataset and 30% test dataset, hyperparameter optimization with grouped 10-fold cross-validation in the training dataset. Models trained on each dataset were evaluated twice: once with the 30% hold-out test dataset from the corresponding training dataset and once with 100% of the respective other dataset. The picture for the standardized dataset is model AF33HAS from the KDEF database [38]. The exemplary picture for the unstandardized dataset symbolizes but is not taken from one of our anonymous participants; the model provided written informed consent.

The machine learning pipeline is depicted in Fig 1 and was identical for both datasets (*standardized* dataset and the *unstandardized* dataset). We first extracted the activity of 20 AUs (see section Datasets) with Noldus FaceReader [43]. During the subsequent preprocessing step, two Action Units with zero variance (AU18 and AU27) were excluded. Both datasets were randomly split into a training (70%) and a test set (30%). As the data is inherently dependent (seven different emotional facial expressions per individual), the random split considered the unique identifier of the individual to prevent biased performance estimates introduced by data leakage (i.e., emotional facial expressions from a person being allocated to the train as well as the test dataset).

During the *training phase*, the hyperparameters of the algorithms were optimized (maximizing kappa, κ) within the 70% training split with a grouped ten-fold cross-validation. Transformation of the data, i.e., scaling and centering, was managed inside the train function of the caret R-package [54] and independently applied to all cross-validation splits to avoid data leakage [57]. Grouping for the cross-validation was again based on the unique identifier of the individual. We decided to assess performance in an independent test set to prevent inflated performance metrics [57] due to optimization errors in cross-validations [58]. Consequently, the final models were trained with the optimal hyperparameters as previously determined by the cross-validation in the 70% training set.

In the *evaluation phase*, we first determined a baseline performance in previously unseen data from the same dataset that was used to train the models, i.e., with the hold-out 30% test set. Models trained with the *standardized* dataset were evaluated in the hold-out 30% test set of this dataset and vice versa; the models trained with the *unstandardized* dataset predicted the unseen hold-out 30% test set of the *unstandardized* dataset. Performance metrics obtained in the hold-out test sets from the same dataset provide an indicator of the model’s ability to predict facial expressions of similar prototypicality. Finally, to determine the influence of the specific training material on the performance, we used the models trained in one dataset to predict the respective other datasets. The models based on the *standardized* dataset were tested with the *unstandardized* dataset and vice versa. We evaluate the classification performance for all models in terms of overall accuracy, kappa (κ), and logLoss, as well as additional binary classification metrics separately for each emotion category: sensitivity (*Sn*), specificity (*Sp*), and the F_1_-score (*F*_*1*_). These binary classification metrics are calculated with a one-versus-all approach [54].

## Results

### Hyperparameter tuning and cross-validation

Optimal hyperparameters for each algorithm were determined with grouped 10-fold cross-validations (maximizing kappa). We tuned the complexity parameter (*cp*) for the decision tree and tested 100 values between 0 and 0.1614 with an interval step of 0.00163. The number of trees (*ntree*) and the variables considered at each split (*mtry*) were optimized for the random forest. We spun a grid with the following values: *mtry* = [1; 18] with steps of 1 and *ntree* = [100; 1000] with intervals of 100. We further optimized the numbers of neurons (*size*) in the single hidden layer of the multi-layer perceptron. In the tuning process, we considered values for *size* between 1 and 18, with intervals of 1. Additional hyperparameters of the algorithms (e.g., *minbucket* the minimum number of observations in a terminal node for the decision tree) are set to their default values; for more details, see *rpart*, *randomForest*, *RSNNS* documentation [50–52].

The optimal hyperparameters for models based on the *standardized* dataset were: *cp* = 0.1207546 (decision tree), *mtry* = 3 and *ntree* = 700 (random forest) and *size* = 11 (multi-layer perceptron). The optimal hyperparameters for models based on the *unstandardized* dataset were: *cp* = 0.006593715 (Decision Tree), *mtry* = 1 and *ntree* = 800 (random forest) and *size* = 17 (multi-layer perceptron). Table 2 presents the performance metrics obtained during the hyperparameter tuning for both datasets.

**Table 2:** Performance metrics in grouped 10-fold cross-validation.

Material	Algorithm	Tune	Accuracy	Kappa	logLoss	Mean Bal. Acc.	Mean Sens.	Mean Spec.
70% Training Data Standardized Dataset	Decision Tree	min	0.771	0.733	0.164	0.869	0.776	0.962
		mean	0.899	0.882	1.124	0.941	0.899	0.983
		max	0.964	0.958	3.369	0.979	0.964	0.994
	Random Forest	min	0.833	0.806	0.104	0.903	0.833	0.972
		mean	0.921	0.908	0.304	0.954	0.922	0.987
		max	1.000	1.000	0.618	1.000	1.000	1.000
	Multi-Layer Perceptron	min	0.750	0.708	0.201	0.857	0.755	0.958
		mean	0.905	0.889	0.501	0.945	0.905	0.984
		max	0.964	0.958	1.493	0.979	0.964	0.994
70% Training Data Unstandardized Dataset	Decision Tree	min	0.643	0.583	0.308	0.792	0.643	0.940
		mean	0.792	0.757	1.505	0.879	0.792	0.965
		max	0.914	0.900	4.171	0.950	0.914	0.986
	Random Forest	min	0.786	0.750	0.343	0.875	0.786	0.964
		mean	0.894	0.877	0.540	0.938	0.894	0.982
		max	1.000	1.000	0.734	1.000	1.000	1.000
	Multi-Layer Perceptron	min	0.750	0.708	0.113	0.854	0.750	0.958
		mean	0.836	0.809	0.848	0.904	0.836	0.973
		max	0.964	0.958	1.487	0.979	0.964	0.994

### Classification performance of standardized dataset models

#### Train standardized–test standardized

**Fig 2:**
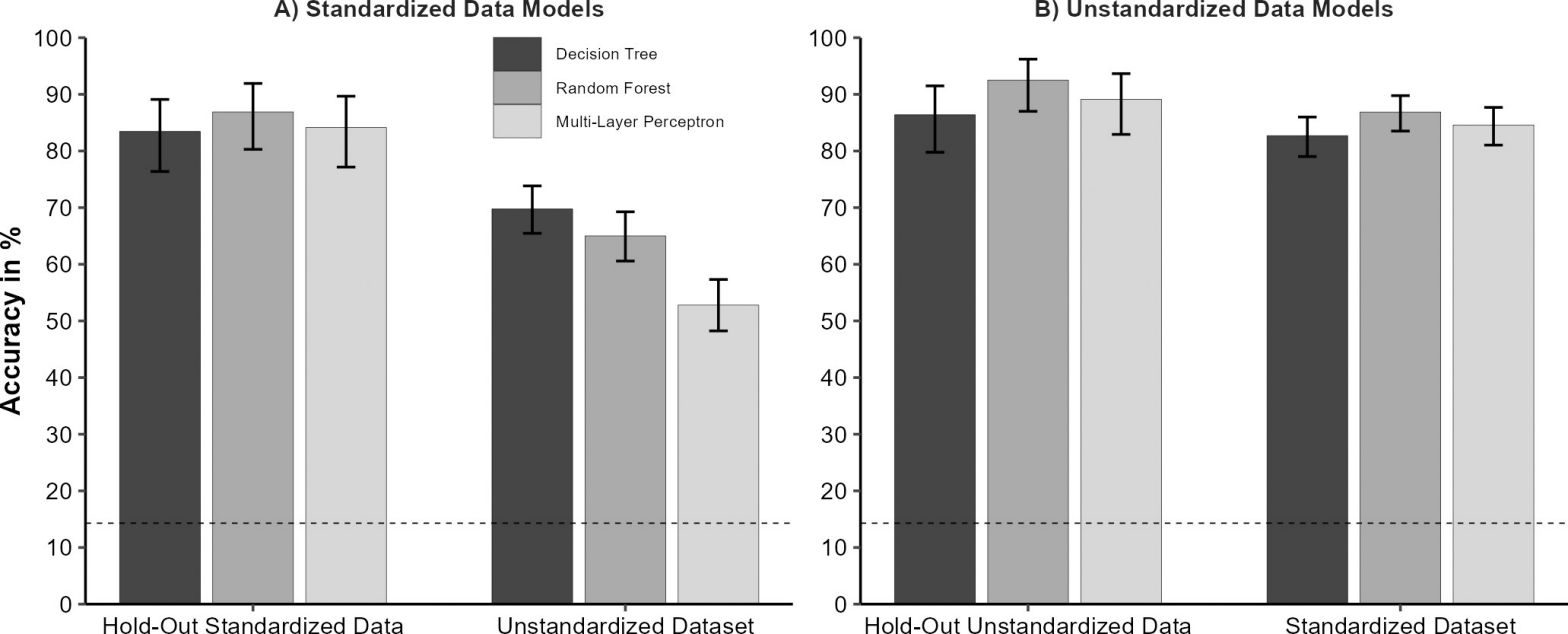
Effect of training material on overall classification performance. *Note*. Overall classification accuracy for the models trained with the *standardized* dataset (Panel A) and the models trained with the *unstandardized* dataset (Panel B); for different test sets on the x-axis. Models were evaluated with either a hold-out test set from the same material or with the respective other dataset. *N*_test_ = 147 in both test sets, *N*_*standardized*_ = 480 in the *standardized* dataset, and *N*_*unstandardized*_ = 483 in the *unstandardized* dataset. Error bars represent the 95% confidence interval [59]. The no-information rate is depicted as a dotted line (14.3%). All accuracies were significantly higher than the no information rate (exact binomial test, all *p*s < .0001, 1-sided).

All models based on the *standardized* dataset predicted the hold-out data from the *standardized* dataset with high accuracy and few misclassifications (Fig 2, Panel A left; decision tree: *Acc* = 83.4%, κ = 0.81, logLoss = 2.71; random forest: *Acc* = 86.9%, κ = 0.85, logLoss = 0.39; multi-layer perceptron: *Acc* = 84.1%, κ = 0.81, logLoss = 0.84). With respect to the binary classification metrics, all emotions were classified with high *F*_*1*_-scores (decision tree: *F*_*1*_ = [76.6%; 97.7%]; random forest: *F*_*1*_ = [78.9%; 100%]; multi-layer perceptron *F*_*1*_ = [78.9%; 97.7%]).

#### Train standardized–test unstandardized

**Fig 3:**
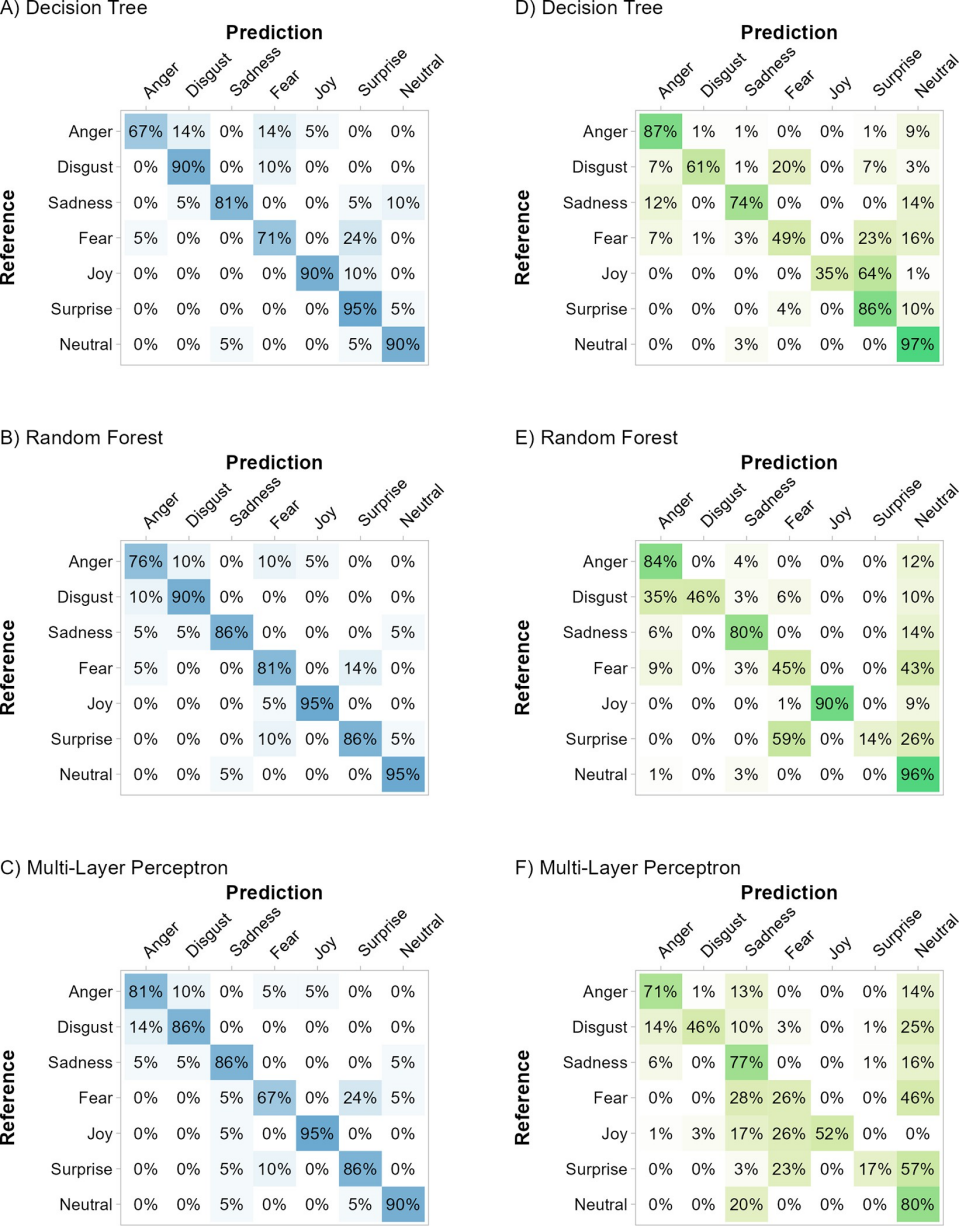
Classification performance of models trained with the standardized dataset. *Note*. Confusion matrices of the models trained with the *standardized* dataset. Left panels A–C performance in the hold-out data from the *standardized* dataset. Right panels D–F performance in the *unstandardized* dataset. The diagonal displays the sensitivity for each basic emotion. Darker color indicates higher frequencies for the specific predicted emotion.

The same models (i.e., trained with the *standardized dataset*) classified the *unstandardized* dataset with substantially impaired accuracy (Fig 2, Panel A, right; decision tree: *Acc* = 69.8%, κ = 0.65, logLoss = 6.71; random forest: *Acc* = 65%, κ = 0.59, logLoss = 1.09; multi-layer perceptron: *Acc* = 52.8%, κ = 0.45, logLoss = 2.71). Substantially impaired classification performance for the models trained with the *standardized* dataset and evaluated in the *unstandardized* dataset is also evident in the low sensitivities for several emotion categories; see the diagonals in the confusion matrices (Fig 3, Panels D-F). All three models trained with the *standardized* dataset show overall reduced accuracies when tested with the less prototypical *unstandardized* dataset (decision tree: -13.6%, random forest: -21.9%, multi-layer perceptron: -31.3%). However, there are some specific differences in the classification performance of the models. For example, the random forest and the multi-layer perceptron models fail to correctly classify surprise in the *unstandardized* dataset (random forest: *Sn* = 14.5%, *Sp* = 100%, *F*_*1*_ = 25.3%; multi-layer perceptron: *Sn* = 17.4%, *Sp* = 99.5%, *F*_*1*_ = 28.9%). In contrast, the more basic decision tree classified surprise well (*Sn* = 85.5%, *Sp* = 84.1%, *F*_*1*_ = 60.8%), but it was less sensitive for joy (*Sn* = 34.8%). The multi-layer perceptron also classified joy with a reduced sensitivity (*Sn* = 52.2%). Common to all three models which were trained with *standardized* data is an increased misclassification of facial expression as neutral. Also, their sensitivities are reduced for fear (all *Sn* ≤ 49.3%) and disgust (all *Sn* ≤ 60.9%).

### Classification performance of unstandardized dataset models

#### Train unstandardized–test unstandardized

**Fig 4:**
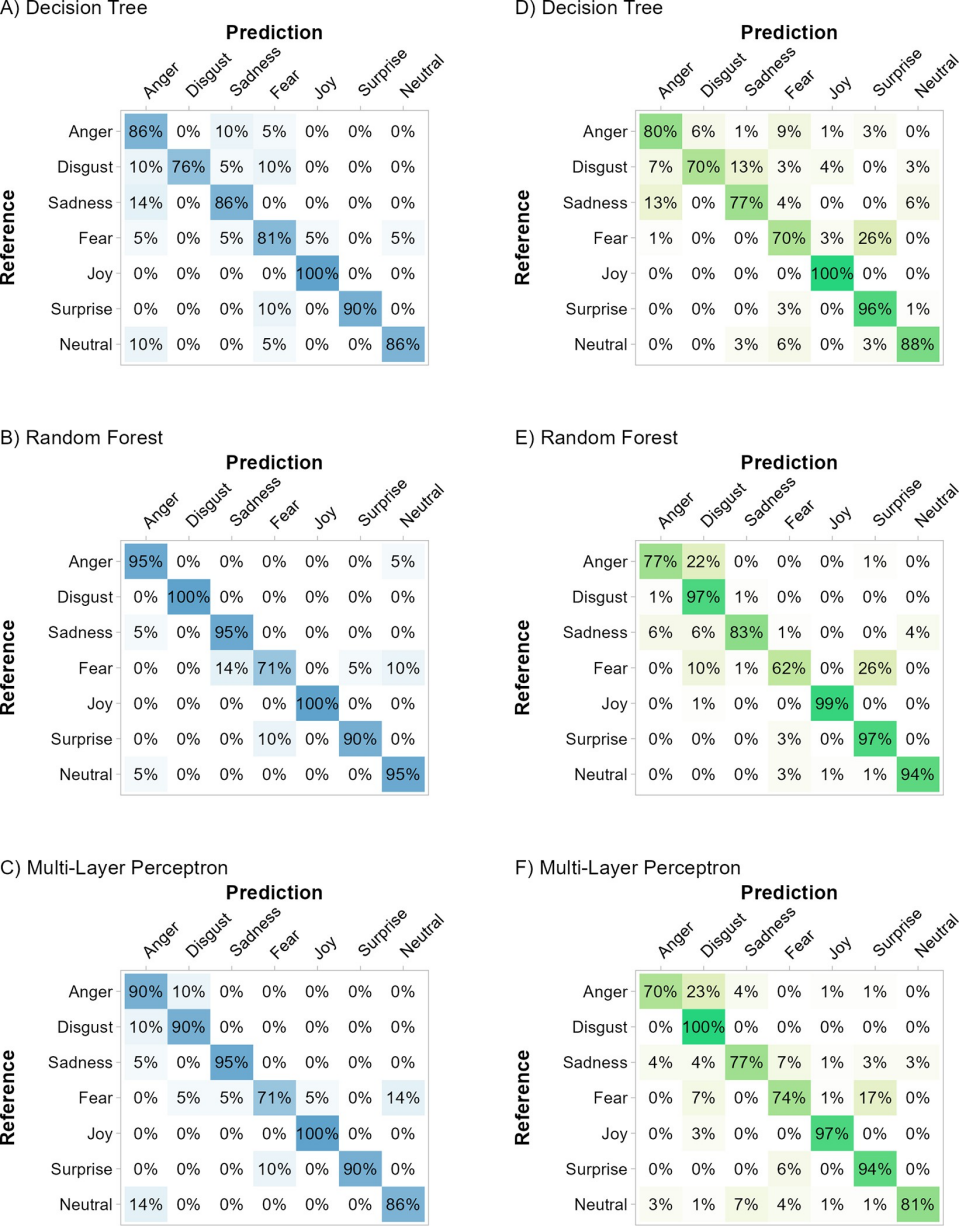
Classification performance of models trained with the unstandardized dataset. *Note*. Confusion matrices of the models trained with the *unstandardized* dataset. Left panels A–C: performance in the hold-out data from the *unstandardized* dataset. Right panels D–F: performance in the *standardized* dataset. The diagonal displays the sensitivity for each basic emotion. Darker color indicates higher frequencies for the specific predicted emotion.

All models based on the *unstandardized* dataset predicted the hold-out data from the *unstandardized* dataset with high accuracy and few misclassifications (see Fig 2, Panel B left; decision tree: *Acc* = 86.4%, κ = 0.84, logLoss = 2.84; random forest: *Acc* = 92.5%, κ = 0.91, logLoss = 0.43; multi-layer perceptron: *Acc* = 89.1%, κ = 0.87, logLoss = 0.54). Correspondingly, the *F*_*1*_-Scores for all emotion categories were high (Fig 4, Panel A-C; decision tree: *F*_*1*_ = [76.6%; 97.7%]; random forest: *F*_*1*_ = [78.9%; 100%]; multi-layer perceptron: *F*_*1*_ = [78.9%; 97.7%]).

#### Train unstandardized–test standardized

The same models classified the *standardized* data with only a slight decrease in the overall classification performance (Fig 2, Panel B right; decision tree: *Acc* = 82.7%, κ = 0.8, logLoss = 1.94; random forest: *Acc* = 86.9%, κ = 0.85, logLoss = 0.5; multi-layer perceptron: *Acc* = 84.6%, κ = 0.82, logLoss = 1.24). The *F*_*1*_-Scores for all three models trained with the *unstandardized* dataset remained high when evaluated with the *standardized* dataset (Fig 4, Panel D-F; decision tree: *F*_*1*_ = [71.6%; 95.7%]; random forest: *F*_*1*_ = [73.5%; 98.5%]; multi-layer perceptron: *F*_*1*_ = [73.5%; 98.5%]). For these models, anger is classified by all *unstandardized* models with slightly reduced sensitivities (all *Sn* ≥ 69.6%). Furthermore, the random forest model performs weakest in the classification of fear (*Sn* = 62.3%, *Sp* = 98.8%, *F*_*1*_ = 73.5%).

## Discussion

Automatic facial coding is a promising new tool to efficiently analyze facial expressions in many research settings and prospectively also in naturalistic settings. However, recent AFC and computer vision developments have heavily relied on highly standardized emotion inventories to train and test the underlying machine learning models. Such prototypical facial expressions are referred to as the gold standard when new software is evaluated [60]. This is problematic because this may not say much about the performance on less intense and less prototypical facial expressions of emotions as they occur in typical laboratory settings or even in the natural environment [19–21]. Accordingly, the present study aimed to investigate the influence of the prototypicality of the picture material (*standardized* vs. *unstandardized* facial expressions) used to train machine learning algorithms on the classification of standardized and non-standardized emotional expressions. This is highly relevant for emotion researchers, who are primarily interested in the valid measurement of naturalistic facial expressions that are less intense and less prototypical [21, 24].

In the present study, machine learning models trained with expressions from either standardized inventories or unstandardized laboratory participants had excellent accuracies when tested on the same source of facial expressions. The recognition rates of the models are comparable to those of human raters and comparable to those reported for models evaluated in standardized research inventories (see Table 1) [60–62]. Accordingly, the models trained with standardized facial expressions predicted expressions from the same dataset with excellent performance metrics. However, these models predicted unstandardized facial expressions of untrained lab-study participants with substantially impaired classification performances, which is well in line with the performance reported by others (accuracy = [.21; .61], *M* = .48, [15, 19, 20]). Hence, models trained with *standardized* dataset did not generalize well to the less prototypical and less intense facial expressions in the *unstandardized* dataset. In contrast, models trained with the *unstandardized* dataset classified the prototypical expressions in the *standardized* dataset again with impressive performance metrics.

This implies that machine learning models benefit from training on less prototypical facial expressions, leading to higher accuracy and increasing the generalizability of the underlying model to more naturalistic facial expressions. Accordingly, such a training approach could improve the ecological validity of AFC systems because typical research participants are untrained and display less intense and less prototypical facial expressions.

While all three models trained with the *standardized* dataset and evaluated in the *unstandardized* dataset show reduced overall accuracy and difficulty detecting the emotions fear and disgust, there are also algorithm-specific deficits for some emotion categories. Compared to the more basic decision tree, the random forest and the multi-layer perceptron, which are considered more sophisticated algorithms, have a pronounced deficit in the classification of surprise. Even though the models trained with *unstandardized* facial expressions generalized well to the standardized dataset, we found lowered sensitivities, e.g., predicting fearful facial expressions. However, this is in line with previous research showing reduced sensitivities for fear not only for AFC systems [7, 20] but also for human emotion recognition [63–65].

### Limitations and future directions

One limitation of our study may be the number of pictures used our machine learning pipeline, which is at the lower end of what is typically recommended for machine learning. Nevertheless, our machine learning models reached very high accuracies in unseen hold-out data, comparable to those in the field [19]. Furthermore, accuracies in the test sets were approaching the accuracies obtained during cross-validation in the training set; thus, there is no serious overfitting. Moreover, accuracies were very well comparable to a now considerable number of studies that relied on models that were trained on larger datasets by the developers of commercial software packages (see Table 1).

However, future studies should test a higher number of pictures and more datasets with further variation of the level of prototypicality in the expressed emotions to establish the generalizability of this result and build up a stock of available datasets with varying degrees of prototypicality in the facial expressions. Correspondingly, our datasets contained expressions from female, relatively young individuals of primarily European descent. Hence, future studies need to replicate our findings on more diverse samples to establish the generalizability of such algorithms regarding gender, age, and ethnicity [66–68].

Three pictures in the standardized dataset had to be excluded as the FaceReader was not able to localize the face. The FaceReader utilizes the viola-jones algorithm for face detection [46]. However, future developments could benefit from adapting newer methods of face detection, e.g., a recent paper described a promising approach of utilizing lightweight convolutional neural networks with ADAM optimization to locate faces in risk situations [69].

Important for future developments in this field could be a look beyond basic emotion categories. While these categories are thought to cover the majority of qualitatively different emotional experiences, there is evidence that facial expressions are much finer-grained and consist of a wider variety of meaningful facial expression categories (e.g.,[2, 70, 71]). In addition to more emotion categories, future studies should also consider new developments in emotion research and evaluate the classification of dimensional representations of emotions, such as valence (e.g., [72, 73]).

Our results demonstrate the relevance of evaluating machine learning algorithms on more naturalistic facial expressions which are different from standardized research inventories. More datasets with lay participants in a laboratory setting are needed to improve the training of underlying machine learning algorithms and optimize the performance of automatic facial coding. With more naturalistic datasets being available to the research community, the generalizability and accuracy of automatic facial coding approaches are expected to increase. Accordingly, the ability of AFC to detect subtle facial responses might improve and approach the sensitivity of other methods, such as facial EMG [74–76]. Higher generalizability and ecological validity of automatic facial coding software will likely lead to broader dissemination of this technology, which can bear ethical implications (e.g., when participants are unaware of being observed) that need to be addressed.

## Conclusions

AFC is an innovative research tool to classify emotional facial expressions effectively. Compared to more traditional manual coding by humans, it is faster and can potentially be implemented in real-time recognition systems (e.g., smartphones [28]). However, AFC is substantially less accurate in unstandardized facial expressions. The present data support the conclusion that this gap in accuracy may be due to the prototypicality of the material used to train algorithms; AFC classifiers, as of today, are typically trained with highly standardized and intense facial expressions. Our results imply that models trained on standardized inventories do not generalize well to unstandardized facial expressions of untrained individuals whom we recruit as laboratory participants. However, models trained with unstandardized expressions performed substantially better when evaluated with frequently used standardized facial expressions from research inventories. Future developments in automatic facial coding will benefit from using less prototypical training materials to increase classification performance in more natural settings. Such a training approach will improve AFC systems’ robustness and ecological validity and contribute to the broader applicability of AFC tools in emotion research.

## Supporting information

S1 Table R-packages for all analyses. (PDF)
