# Supplementary material for: Training machine learning algorithms for automatic facial coding: The role of emotional facial expressions’ prototypicality
Source: PLoS One. 2023 Feb 10;18(2):e0281309. doi: 10.1371/journal.pone.0281309 (PMC9916590; doi:10.1371/journal.pone.0281309)
Supplement: S1 Table — (PDF) [file pone.0281309.s001.pdf]

S1 Table. R-Packages for all analyses

Package	Version	Maintainer	Citation
caret	6.0.92	Max Kuhn <mxkuhn@gmail.com>	Kuhn, M. (2022). *caret: Classification and Regression Training*. https://github.com/topepo/caret/
conflicted	1.1.0	Hadley Wickham <hadley@rstudio.com>	Wickham, H. (2021). *conflicted: An Alternative Conflict Resolution Strategy*. https://cran.r-project.org/package=conflicted
datscience	0.2.4	Björn Büdenbender <b_buedenbender@yahoo.de>	Büdenbender, B. (2022). *datscience: Dataanalysis and Science Utility Functions*. https://buedenbender.github.io/datscience/
dplyr	1.0.9	Hadley Wickham <hadley@rstudio.com>	Wickham, H., François, R., Henry, L., & Müller, K. (2022). *dplyr: A Grammar of Data Manipulation*. https://cran.r-project.org/package=dplyr
forcats	0.5.1	Hadley Wickham <hadley@rstudio.com>	Wickham, H. (2021). *forcats: Tools for Working with Categorical Variables (Factors)*. https://cran.r-project.org/package=forcats
foreign	0.8.82	R Core Team <R-core@R-project.org>	R Core Team. (2022). *foreign: Read Data Stored by Minitab, S, SAS, SPSS, Stata, Systat, Weka, dBase, ...* https://svn.r-project.org/R-packages/trunk/foreign/
ggplot2	3.3.6	Thomas Lin Pedersen <thomas.pedersen@rstudio.com>	Wickham, H., Chang, W., Henry, L., Pedersen, T. L., Takahashi, K., Wilke, C., Woo, K., Yutani, H., & Dunnington, D. (2022). *ggplot2: Create Elegant Data Visualisations Using the Grammar of Graphics*. https://cran.r-project.org/package=ggplot2
ggthemes	4.2.4	Jeffrey B. Arnold <jeffrey.arnold@gmail.com>	Arnold, J. B. (2021). *ggthemes: Extra Themes, Scales and Geoms for ggplot2*. https://github.com/jrnold/ggthemes
glue	1.6.2	Jennifer Bryan <jenny@rstudio.com>	Hester, J., & Bryan, J. (2022). *glue: Interpreted String Literals*. https://cran.r-project.org/package=glue
gridExtra	2.3	Baptiste Auguie <baptiste.auguie@gmail.com>	Auguie, B. (2017). *gridExtra: Miscellaneous Functions for "Grid" Graphics*. https://cran.r-project.org/package=gridExtra
iml	0.11.0	Christoph Molnar <christoph.molnar@gmail.com>	Molnar, C., Bischl, B., & Casalicchio, G. (2018). iml: An R package for Interpretable Machine Learning. *JOSS*, *3*(26), 786. https://doi.org/10.21105/joss.00786

Package	Version	Maintainer	Citation
lattice	0.20.45	Deepayan Sarkar <deepayan.sarkar@r-project.org>	Sarkar, D. (2008). *Lattice: Multivariate Data Visualization with R*. Springer. http://lmdvr.r-forge.r-project.org
mlbench	2.1.3	Friedrich Leisch <Friedrich.Leisch@R-project.org>	Leisch, F., & Dimitriadou., E. (2021). *mlbench: Machine Learning Benchmark Problems*. https://cran.r-project.org/package=mlbench
psych	2.2.5	William Revelle <revelle@northwestern.edu>	Revelle, W. (2022). *psych: Procedures for Psychological, Psychometric, and Personality Research*. https://personality-project.org/r/psych/
purrr	0.3.4	Lionel Henry <lionel@rstudio.com>	Henry, L., & Wickham, H. (2020). *purrr: Functional Programming Tools*. https://cran.r-project.org/package=purrr
randomForest	4.7.1	Andy Liaw <andy_liaw@merck.com>	Breiman, L., Cutler, A., Liaw, A., & Wiener, M. (2022). *randomForest: Breiman and Cutler's Random Forests for Classification and Regression*.
Rcpp	1.0.9	Dirk Eddelbuettel <edd@debian.org>	Eddelbuettel, D., Francois, R., Allaire, J. J., Ushey, K., Kou, Q., Russell, N., Ucar, I., Bates, D., & Chambers, J. (2022). *Rcpp: Seamless R and C++ Integration*. https://cran.r-project.org/package=Rcpp
readr	2.1.2	Jennifer Bryan <jenny@rstudio.com>	Wickham, H., Hester, J., & Bryan, J. (2022). *readr: Read Rectangular Text Data*. https://cran.r-project.org/package=readr
rpart	4.1.16	Beth Atkinson <atkinson@mayo.edu>	Therneau, T., & Atkinson, B. (2022). *rpart: Recursive Partitioning and Regression Trees*. https://cran.r-project.org/package=rpart
RSNNS	0.4.14	Christoph Bergmeir <c.bergmeir@decsai.ugr.es>	Bergmeir, C. (2021). *RSNNS: Neural Networks using the Stuttgart Neural Network Simulator (SNNS)*. https://github.com/cbergmeir/RSNNS
scales	1.2.0	Hadley Wickham <hadley@rstudio.com>	Wickham, H., & Seidel, D. (2022). *scales: Scale Functions for Visualization*. https://cran.r-project.org/package=scales
stringr	1.4.0	Hadley Wickham <hadley@rstudio.com>	Wickham, H. (2019). *stringr: Simple, Consistent Wrappers for Common String Operations*. https://cran.r-project.org/package=stringr
tibble	3.1.7	Kirill Müller <krlmlr+r@mailbox.org>	Müller, K., & Wickham, H. (2022). *tibble: Simple Data Frames*. https://cran.r-project.org/package=tibble

Package	Version	Maintainer	Citation
tictoc	1.0.1	Sergei Izrailev <sizrailev@collective.com>	Izrailev, S. (2021). *tictoc: Functions for Timing R Scripts, as Well as Implementations of Stack and List Structures*. https://github.com/collectivemedia/tictoc
tidyr	1.2.0	Hadley Wickham <hadley@rstudio.com>	Wickham, H., & Girlich, M. (2022). *tidyr: Tidy Messy Data*. https://cran.r-project.org/package=tidyr
tidyverse	1.3.1	Hadley Wickham <hadley@rstudio.com>	Wickham, H. (2021). *tidyverse: Easily Install and Load the Tidyverse*. https://cran.r-project.org/package=tidyverse
